# The Spectrum of Mitochondrial Mutation Differs across Species

**DOI:** 10.1371/journal.pbio.0060213

**Published:** 2008-08-26

**Authors:** Kristi L Montooth, David M Rand

## Abstract

Mitochondrial DNA mutation rates have now been measured in several model organisms. The patterns of mutation are strikingly different among species and point to modulation of mutation-selection balance in the evolution of nucleotide composition.

Mutations are ubiquitous, and many arise during the very process of replicating and transmitting genomes. This process is the source of the genetic variation that provides the raw material for both evolutionary novelty and human disease. Mutation rates are known to vary among nucleotides, across genomic regions, and between taxa. It is conventional wisdom that animal mitochondrial DNA (mtDNA) is one genomic region that has a particularly high mutation rate. Until recently, this high rate of mutation has been predominantly inferred from high levels of mitochondrial sequence divergence between species. However, the apparently simple process of mutation and sequence divergence is intriguingly complex in mitochondria, due to the unique biology of these extrachromosomal genomes.

Unlike nuclear DNA, where a new mutation arises on one of four possible DNA strands that can be passed to a diploid offspring, a new mtDNA mutation exists on one of many thousands of mtDNA strands that might (or might not) get incorporated into an egg. With a complex cellular pedigree of mtDNA molecules per mitochondrion, mitochondria per egg cell, egg cells per female, and an even more complex pedigree of females per population, it is a complicated path from mtDNA mutation to fixed mtDNA difference between species [1]. The basic biology of this problem was sketched out more than 30 years ago in a pioneering study of mtDNA sequence variation in sheep and goats by Upholt and Dawid [2]. They recognized the clonal nature of mtDNA inheritance, the random drift process acting on mutations within cytoplasms, and the likelihood that mutations may contribute to variation within species but not become fixed substitutions between species. In short order, mtDNA became a powerful tool of population and evolutionary biologists when it was realized that the rapid rate of mitochondrial mutation and evolution was useful for evolutionary inference [3,4]. In the mid-1980s, mtDNA mutations became candidates for human disease as several papers attributed a variety of disorders to specific point mutations and deletions in the mitochondrial genome [5–7].

In the ensuing years, mutation in the mitochondrial genome has been studied intensively by two different camps: evolutionary biologists, who assumed that mtDNA mutations had no significant functional effects and would serve as reliable neutral markers, and molecular and cell biologists, who saw mtDNA mutations as an underappreciated source of human pathologies. However, it is becoming increasingly popular to apply evolutionary models to problems in mitochondrial disease [8,9] and to examine molecular mechanisms of mutation among strains of model organisms that have been allowed to mutate and evolve in the lab. What we are learning after three decades of extensive study is that the spectrum of mitochondrial mutations varies widely across taxa, with important consequences for the mutation-selection balance maintaining nucleotide composition. However, a new flurry of papers quantifying mitochondrial mutation rates in mutation accumulation studies across model organisms is showing us just how much we still have to learn about mtDNA mutation, variation, and evolution.

## Measuring Mutation Without the Filter of Natural Selection

The problem of inferring mutation rates from sequence divergence between species is that this approach largely detects only those mutations that have no detrimental effect on organismal survival or reproduction (i.e., neutral mutations). Most new mutations will be lost, and this can be an accident of genetic sampling or a consequence of deleterious effects of mutations. To accurately estimate true mutation rates, and not observed substitution rates, one must identify novel variants shortly after they are generated. There are two approaches to this problem. One can capture daughter strands after very few rounds of DNA replication, or one can culture organisms in a manner that reduces the strength of the selective filter. A recent study in mice employed these approaches by sequencing many complete mtDNAs in offspring from mothers carrying a mutation for the proofreading activity of mtDNA polymerase [10]. As expected, these “mutator mice” showed very high levels of mtDNA mutation and established that purifying selection removes new mutations in as few as two generations of transmission. This study confirmed earlier reports that showed a 10-fold difference between mtDNA mutation and substitution rates [11–13].

A more common method of studying mutation is to generate mutation accumulation (MA) lines in the lab. MA lines are cultured using the minimum number of founding parents per generation to minimize the removal of deleterious mutations by natural selection. In an asexual organism like Caenorhabditis elegans, replicate MA lines are perpetuated using single individuals, reducing the effective breeding population size to one. In obligate sexual species such as Drosophila two parents are needed, but the effective population size approaches one if single-pair full-sib mating is followed for many generations. Natural selection can only filter out mutations with fitness effects on the order of the reciprocal of the effective population size (1/*N*_e_). When *N*_e_ approaches one, as it does in MA lines, selection against new deleterious mutations approaches zero. Reducing *N*_e_ in this manner increases the effects of genetic drift and renders even strongly deleterious mutations effectively neutral. In theory only lethal mutations will be eliminated during the creation of MA lines, but in practice lines carrying strongly deleterious alleles become hard to maintain, or show delayed development, and are often lost. In short, MA lines turn down the knob on selection, providing a window into the spectrum of mutations that arise before they are filtered by natural selection.

In 2000, Denver and colleagues [12] sequenced nearly complete mtDNAs from MA lines of C. elegans, revealing that the mtDNA mutation rate was about ten times higher than the observed mtDNA substitution rate between species. The majority of point mutations observed among the MA lines were amino acid–altering changes. This is in sharp contrast to the divergence between species, which is largely comprised of synonymous change. The ratio of nonsynonymous to synonymous mutations was 9:6 among the MA lines, but was only 3:25 between two wild isolates of C. elegans. These results demonstrate the strong effect of natural selection, not only in decreasing the overall number of mutations that accumulate between species, but specifically in filtering out nonsynonymous mutations before they become established in natural populations.

In the current issue of *PLoS Biology*, a new study of Drosophila melanogaster MA lines uncovers several novel features of the mtDNA mutation process [14]. Again, the pervasive effects of purifying selection are evident. The ratio of nonsynonymous to synonymous mutations appearing in the MA lines was 24:1 [14], but only 10:36 between two strains of D. melanogaster [15]. The evidence for strong purifying selection removing mtDNA mutations is now very solid and remarkably repeatable across taxa [12,10,14]. What is unexpected from the new studies is the striking difference in the patterns of mutation biases that are now evident among different organisms.

## A Muddle of Mutation across Taxa

The mitochondrial genomes of yeast [16], C. elegans [12], and Drosophila [14] all exhibit elevated mutation rates relative to their nuclear counterparts. The magnitude of the ratio between mitochondrial and nuclear mutation rates varies across taxa, with yeast, C. elegans, and mammals having particularly high ratios [16]. However, this ratio depends critically on how per-generation estimates of the mutation rate are adjusted for the number of DNA replication events during germ cell development. For biparental nuclear genomes, the mutation rate is typically scaled by the number of germ-line cell divisions in each sex. This scaling is also done in estimating mtDNA mutation rates, but the number of germ cell divisions differ between males and females. This difference varies across species [17], which could alter the rate ratio for mtDNA versus nuclear DNA. A greater conundrum is how one should correct for the number of mtDNA replication events during germ cell development. An elevated rate of mitochondrial mutation could result from an increased number of mtDNA replication events relative to the nuclear DNA during germ line development (e.g., [18]), differences in the suite of DNA repair mechanisms [19], the extensive time that the lagging strand of the mtDNA is exposed as a single strand during replication (reviewed in [20]), and the high potential for oxidative damage to DNA in the mitochondrion [21]. Until we have better estimates of these factors, it will be difficult to know (1) the true extent of variation across taxa in the ratio of mtDNA to nuclear mutation rates and (2) how much of the elevated rate of mitochondrial to nuclear mutation is due to elevated rates of mutation per replication event and how much can be attributed to the simple fact that the mtDNA may experience many more rounds of replication and mutation per germ-cell division than do nuclear genomes.

**Figure 1:**
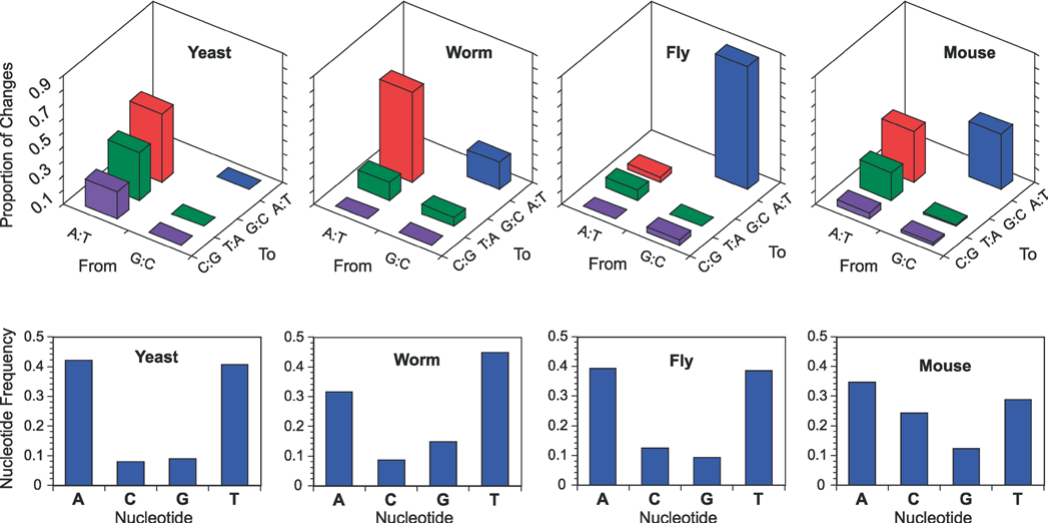
Mutation Patterns and Base Composition among Mitochondrial Genomes The two transitions are in red and blue, while the four possible transversions are in purple and green. Mutation spectrum data are from MA lines of Saccharomyces cerevisiae [16], C. elegans [12], and D. melanogaster [14], as well as mitochondrial mutator strains of Mus musculus [10]. Nucleotide frequencies are from complete mtDNA sequences from each species (Drosophila data exclude the A+T-rich D-loop region).

What is more clear from the MA data is that the process of mitochondrial mutation is highly asymmetric between nucleotides (e.g., guanine → adenine # adenine → guanine), and this asymmetry is strikingly taxon-specific (Figure 1). Certain base pairs are more susceptible to specific mutations, particularly when DNA is single-stranded (for a review see Chapter 6 in [22]). The spontaneous deamination of cytosine causes cytosine:guanine (C:G) base pairs to mutate to thymine:adenine (T:A) base pairs (a transition), while the oxidative conversion of guanine to 8-oxo-guanine results in the modification of G:C base pairs to T:A or A:T base pairs to C:G (both transversions). Adenines in single-stranded DNA can be converted to hypoxanthine, resulting in transitions from A:T base pairs to G:C. It remains unclear why these modifications should occur at different rates across taxa. However, the taxon-specific asymmetries in the mitochondrial mutation process that are evident in the recent MA data (Figure 1) indicate that the core process of mutation changes dramatically along metazoan (and yeast) mtDNA lineages. This taxon-specificity of the mutation landscape has important implications for the maintenance of nucleotide composition in mitochondrial genomes.

## Maintaining Nucleotide Composition in a Rain of Biased Mutation

If mutation were the only force maintaining a stable equilibrium base composition, we would expect that the numbers of reciprocal mutations observed in MA lines would be balanced (e.g., G → A = A → G). A biased mutation pressure that results in unequal numbers of reciprocal mutations should lead to directional shifts in base composition when left unchecked. The data emerging from MA studies reveal that the number of reciprocal mutations in the mtDNA are anything but balanced, and suggest that other forces oppose the mutation pressure in order to maintain stable equilibrium nucleotide composition.

In the new Drosophila study [14], a strongly biased mutation pressure was observed, with 23 of 28 mutations changing from G:C to A:T with only a single reciprocal change from A:T to G:C. This is striking, given that G:C base pairs are far outnumbered by A:T base pairs in the D. melanogaster mtDNA, resulting in a high rate of mutation per G:C relative to the rate per A:T. In other words, mutation in the D. melanogaster mtDNA occurs almost exclusively (25/28) at the more rare G:C nucleotide pairs, and in a direction that favors an increasingly A+T-rich genome. The D. melanogaster MA lines provide some insight into what force may be balancing the asymmetrical mutation pressure in the mtDNA. While 23 of the 28 observed mutations were from G:C to A:T, nearly all of these changes were nonsynonymous and would likely be removed from populations by the filter of natural selection. In Drosophila, it appears that natural selection to preserve amino acid sequence maintains G and C nucleotides in the mtDNA. This is consistent with the overall greater G+C-content at second relative to third codon positions in the Drosophila mtDNA. Lynch (2007) [22] has proposed that a balance between mutation and gene conversion (which tends to be G+C-biased) can explain much of the variation in nucleotide composition observed across nuclear genomes. Gene conversion may occur in mitochondrial genomes [23] and could provide an additional force that balances a mutation pressure that is strongly biased towards A+T in Drosophila mtDNA.

But other MA data reveal that the mutation-selection balance operating in yeast [16] and C. elegans [12] differs from that in the Drosophila mtDNA. In yeast and C. elegans, the majority of observed mitochondrial mutations were from A:T to G:C, with no observed reciprocal G:C to A:T mutations observed in yeast (Figure 1). Yet despite this reversal in the pattern of mutation, all three organisms maintain similarly A+T-rich mtDNA (Figure 1.) In yeast and C. elegans, the probability of a mutation occurring is more consistent with the frequency at which the mutated nucleotide occurs in the mtDNA. However, the unequal number of reciprocal mutations (A:T → G:C >> G:C → A:T) suggests that in yeast and C. elegans there must be some force acting to maintain an A+T-rich base composition in the face of a G+C-biased mutation pressure. This difference among taxa is surprising and motivates further study to understand how and why the mutation-selection balance reverses along mitochondrial lineages.

When mutation probabilities are biased across nucleotides, as appears to be the case in the mtDNA, shifts in equilibrium nucleotide composition will change the overall per-base-pair genomic mutation rate. This is because the scope for mutation to occur is changing as nucleotide composition changes. For example, if mutation occurs almost exclusively at G:C pairs, and a genome were to adopt a new equilibrium nucleotide content with fewer G+C nucleotides while the mutation probabilities remained the same, the new overall per-base-pair genome mutation rate would decrease, as there are fewer possible G+C nucleotides available at which mutation could occur. The nearly exclusive change at G:C base pairs in the Drosophila mtDNA coupled with a low G+C content may generate a low overall mtDNA mutation rate and may contribute to its decreased ratio of mitochondrial to nuclear mutation rates relative to yeast and C. elegans [16]. The entwined nature of nucleotide composition and mutation provides a challenge in deciphering the underlying cause of variation in mutation spectrum observed across yeast, worm, flies, and mouse.

## Heteroplasmy: Catching Mitochondrial Mutation in the Act

The new Drosophila study [14] has capitalized on the unique biology of mutation in mtDNA to provide insights into the transmission process of the mitochondrial genome. When a mutation occurs in mtDNA it generates a condition called heteroplasmy, or a mixed cytoplasm of different genotypes of mtDNA molecules. This new mutation will drift in frequency as the population of mtDNAs replicates within different mitochondria and as different mitochondria experience the sampling process of transmission that occurs during cytokinesis at cell division in the germ line. The length of time (in cell generations) that it takes for a mutation to reach fixation in a germ line depends on the effective population size of mtDNA molecules that produce daughter mtDNA molecules. This “effective number of mitochondria” likewise determines the number of generations that a heteroplasmic germ line will persist. The vast majority of the mutations detected in the Drosophila MA lines were in heteroplasmic condition (see Table 3 of [14]). The distribution of new-mutant frequencies characterizes this drift process and can be used to estimate the mitochondrial effective population size through the germ line. Haag-Liautard and colleagues [14] use a maximum likelihood procedure to obtain an estimate of 13–42 as the effective number of mitochondria. This is more than 10-fold smaller than previous studies that have measured the drift in frequency of mtDNA length variants among heteroplasmic lines of Drosophila [24]. The discrepancy between these two studies may lie in different estimates of the number of germ cell generations per animal generation (see also [24] and [17]).

This intracellular phase of polymorphism is a critical—and poorly understood—phase of mitochondrial genome transmission. When mixed populations of mtDNAs occur in the same mitochondrion, other genetic events could occur, hidden by our ignorance of how mitochondria actually populate the cytoplasm. Heteroplasmic cytoplasms are “heterozygous” and thus allow for the signature of recombination and gene conversion to leave a mark on mtDNA. Both processes have been implicated in several studies [23,25,26], and gene conversion could lead to a directional shift in mtDNA haplotype frequencies. Because any new mitochondrial mutant must run the gauntlet of cellular and molecular events in the germ line in order to ultimately fix in a population, we need to know much more about the population dynamics of mtDNA in germ line cytoplasms in a diversity of organisms. It remains quite possible that the striking differences across taxa in the mutation process and the presumed selective forces that balance this pressure lie hidden in the biology that takes place in these critical divisions of the germ line.

## Conclusion

The wealth of recent data from MA experiments across taxa provides a picture of the mutation spectrum that is far from evolutionarily constant. Mitochondrial genomes from yeast, worm, flies, and mouse experience qualitatively different mutational input, yet maintain qualitatively similar nucleotide content through a mutation-conversion-selection balance that remains to be explained. While pervasive positive selection has recently been posited for the mtDNA [27], this theory remains controversial [28]. The wealth of new MA data suggests that background selection [29] must have strong effects on the evolution of a completely linked mitochondrial genome that experiences extensive purifying selection to remove mutations. Far from being a neutral molecule, the mitochondrial genome appears to have ample scope to be shaped by negative as well as positive selection.

## Abbreviations

MA: mutation accumulation
mtDNA: mitochondrial DNA
